# Candidate Genes for IgA Nephropathy in Pediatric Patients: Exome-Wide Association Study

**DOI:** 10.3390/ijms242115984

**Published:** 2023-11-05

**Authors:** Anastasiia A. Buianova, Mariia V. Proskura, Valery V. Cheranev, Vera A. Belova, Anna O. Shmitko, Anna S. Pavlova, Iuliia A. Vasiliadis, Oleg N. Suchalko, Denis V. Rebrikov, Edita K. Petrosyan, Dmitriy O. Korostin

**Affiliations:** 1 Center for Precision Genome Editing and Genetic Technologies for Biomedicine, Pirogov Russian National Research Medical University, Ostrovityanova Str., 1, p. 1, 117513 Moscow, Russia; feroval@yandex.ru (V.V.C.); verusik.belova@gmail.com (V.A.B.); annashmi97@gmail.com (A.O.S.); pavlova.a.s@gmail.com (A.S.P.); julia.vasiliadis@gmail.com (I.A.V.); olegsuchalko@gmail.com (O.N.S.); ncagip4@gmail.com (D.V.R.); d.korostin@gmail.com (D.O.K.); 2 Nephrology Department, Russian Children’s Clinical Hospital, Leninsky Prospect 117, 119571 Moscow, Russia; md.proskura@gmail.com (M.V.P.); ed3565@yandex.ru (E.K.P.)

**Keywords:** autoimmune disease, exome sequencing, genetic predisposition to disease, IgA nephropathy, pediatrics

## Abstract

IgA nephropathy (IgAN) is an autoimmune disorder which is believed to be non-monogenic. We performed an exome-wide association study of 70 children with IgAN and 637 healthy donors. The HLA allele frequencies were compared between the patients and healthy donors from the bone marrow registry of the Pirogov University. We tested 78,020 gene markers for association and performed functional enrichment analysis and transcription factor binding preference detection. We identified 333 genetic variants, employing three inheritance models. The most significant association with the disorder was observed for rs143409664 (*PRAG1*) in the case of the additive and dominant models (P_BONF_ = 1.808 × 10^−15^ and P_BONF_ = 1.654 × 10^−15^, respectively), and for rs13028230 (*UBR3*) in the case of the recessive model (P_BONF_ = 1.545 × 10^−9^). Enrichment analysis indicated the strongly overrepresented “immune system” and “kidney development” terms. The HLA-DQA1*01:01:01G allele (*p* = 0.0076; OR, 2.021 [95% CI, 1.322–3.048]) was significantly the most frequent among IgAN patients. Here, we characterized, for the first time, the genetic background of Russian IgAN patients, identifying the risk alleles typical of the population. The most important signals were detected in previously undescribed loci.

## 1. Introduction

IgA nephropathy (IgAN) is one of the most common primary glomerulonephritides in the world, both in adults and children [1]. Although it has been extensively studied for more than half a century, it still remains the leading cause of end-stage kidney disease (ESKD) [2]. The clinical presentation of the disease is highly variable: from painless microhematuria to rapidly progressing glomerulonephritis and ESKD [3,4,5,6,7]. Between 20% and 40% of cases of IgAN progression to ESKD within 20 years from the onset require renal replacement therapy [8,9,10,11].

IgA nephropathy is diagnosed through kidney biopsy examination. This shows the deposition of IgA-containing immune complexes in the mesangium inducing mesangial cell proliferation and extracellular matrix accumulation [1]. At present, there are two basic views on the causes of the disease. Several groups suggest that IgAN is an autoimmune disease, leading to the antibody-mediated destruction of the glomerular basement membrane. IgAN pathogenesis is complex and likely to involve several different pathways forming a complex network where infections may play a triggering role. Though, in some cases, IgA nephropathy can precede the infection that induces a dysregulated immune response, IgA nephropathy itself is not an infectious disease [12].

Furthermore, in IgAN, the glycosylation of O-linked glycans in the hinge region of IgA1 is disrupted, resulting in high blood levels of circulating galactose-deficient IgA1 (Gd-IgA1) and its abnormal clearance [13]. Gd-IgA1 has been shown to be highly heritable; therefore, the link between IgAN and Gd-IgA1 and its role in IgAN pathogenesis might provide novel insights into the pathogenic processes involved in IgAN [14]. Levy M et al. suggested a possible contribution of genetic predisposition to IgAN [15]. Their hypothesis is based on a certain geographical prevalence of IgAN. Based on kidney biopsy, IgA nephropathy was diagnosed in 20% and 40% of children with glomerular diseases in Europe and Asia, respectively, whereas it was not frequently registered in the African population [7,16].

The presence of genes involved in immunity against intestinal pathogens is thought to be responsible for the high incidence of IgA nephropathy in Asia. Certain loci are associated with the risk of inflammatory bowel disease (IBD), the state of the intestinal epithelial barrier, and response to mucosal pathogens. Genetic predisposition strongly correlates with helminth diversity, suggesting a possible role for host–intestinal pathogen interactions in IgAN’s geographic variations [17]. Familial cases of IgAN are also known and have been described, supporting the genetic role in the disease etiology [18,19,20,21,22].

The role of gene-candidates in IgAN development was estimated through genome-wide association study (GWAS). In familial forms, a high risk of the disease was found to correlate with the following specific loci: 6q22–23 (*IGAN1*)*,* 4q26–31 (*IGAN2*)*,* and 17q12–22 (*IGAN3*) [23,24]. In East Asia, the associated proteins contribute to adaptive and innate immunity, IgA1 glycosylation, and the renin–angiotensin system [25], including haplotypes *HLA-DQ* and *HLA-DR*: HLA-DRB1*14:05:01 (belonging to DR*14), HLA-DRB1*03:01:01, HLA-DRB1*04, and HLA-DQB1*03:01 [26,27,28]. Xia YF et al. showed the Megsin gene (*SERPINB7*) to be a major factor determining predisposition to the disease and its progression in the Chinese population [29,30]. Twenty-four candidate genes associated with IgAN were analyzed to identify their interactions. The cooperation between *C1GALT1-330G/T* (rs1008898) and *IL5RA31+197A/G* (rs340833) was found to be statistically significant (*p* < 0.001) for IgAN pathogenesis [31], whereas the combination of *P-selectin-2441A/G* with *CD14-159C/T* was associated with gross hematuria in IgAN patients. Moreover, the interaction of *TGF-b1-509T/C, P-selectin-2441A/G*, and *MCP-1-2518A/G* was found to influence the glomerular crescent formation [32]. The interaction between two key genes, *C1GALT1* and *ST6GALNAC2*, was also shown to affect the susceptibility to IgAN and disease progression [33]. In the UK population, a significant association with IgAN was detected in chromosome 6 in the region of the major histocompatibility complex (MHC) (*p* = 1 × 10^−9^) [34]. Five loci associated with susceptibility to IgAN were identified in Chinese patients: three distinct loci in the MHC region, a common deletion of the *CFHR1* and *CFHR3* 1q32 loci, and locus 22q12 (*HORMAD2*) [35]. In 2014, Kiryluk et al. revealed six haplotypes associated with a high risk of IgAN: already known variants in *ITGAM, ITGAX, VAV3,* and *CARD9* genes, as well as two new haplotypes, *HLA-DQB1* and *DEFA* [17]. Ming Li et al. identified genes responsible for susceptibility to the disease and associated with IgAN, located in 17p13 and 8p23 [36], including the tumor necrosis factor (*TNFSF13*) and α-defensin (*DEFA*). rs660895 (*HLA-DRB1*) was found to correlate with the IgA serum level and proteinuria level [37]. The novel genes associated with IgAN included *ST6GAL1* at 3q27.3, *ACCS* at 11p11.2, and *ODF1-KLF10* at 8q22.3. The *ITGAX–ITGAM* (16p11.2) association was confirmed as being moderately replicated, and previously observed genes *VAV3* (1p13) and *CARD9* (9q34) were detected as well.

Given that the genetic aspects of IgAN have previously demonstrated high heterogeneity in terms of the identified associations, we performed an exome-wide association study (EWAS) employing our own clinical samples.

## 2. Results

### 2.1. Clinical Characteristics

The clinical characteristics of 70 IgAN patients are summarized in Table A1.

We found no significant effects of sex on the clinical features based on the one-way analysis of variance (ANOVA) test (for serum creatinine level: F(1,68) = 0.349, *p* = 0.557; for eGFR: F(1,68) = 0.733, *p* = 0.395; for proteinuria level: F(1,68) = 0.02, *p* = 0.889; for median blood pressure: F(1,68) = 0.331, *p* = 0.567). However, symptoms at the debut and sex were influenced by the age of onset, and their interaction had a statistically significant effect (two-way ANOVA: F(1,66) = 6.834; *p* = 0.011). The 637 control samples had an even distribution of sexes, with 52.04% males and 47.96% female.

### 2.2. Morphological Analysis

**Figure 1:**
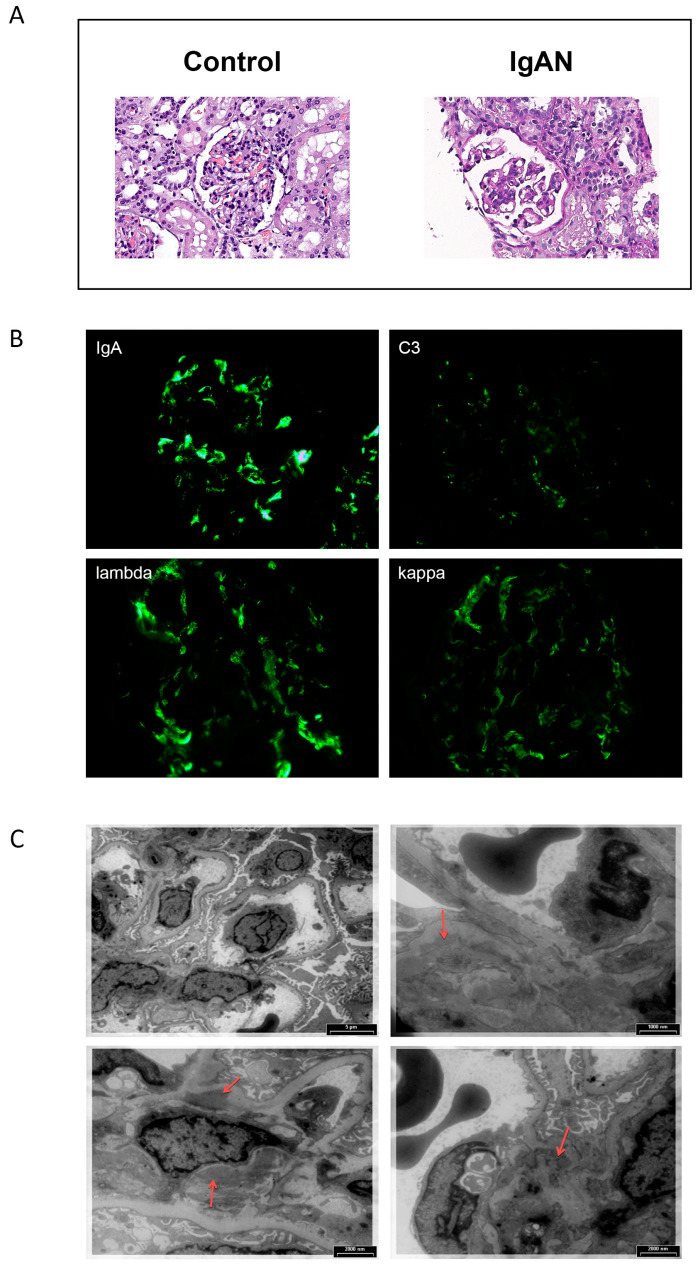
Renal biopsy of a typical patient with IgA nephropathy (M1E0S1T0-C0, according [38]). Hematoxylin-eosin stain (magnification ×400), compared with control sample (**A**). Immunofluorescence microscopy, with staining for IgA, C3, lambda, and kappa (magnification ×400) (**B**). Electron microscopy. Red arrows indicate paramesangial deposits (**C**).

Morphological data of kidney biopsy samples typical of their diagnosis are presented in Figure 1.

In the histological sample, matrix expansion and mesangial hypercellularity (up to 10 cells per a mesangial region) are presented (Figure 1A). According to immunofluorescence, the following can be observed: IgA, kappa, lambda in mesangium—+++. C3—++. IgG, IgM, C1q, fibrinogen—negative. (Figure 1B). The electron microscopy study reveals mild mesangial proliferation (4–6 nuclei) and multiple large paramesangial deposits (Figure 1C).

### 2.3. Description of the Sequencing Data

The main quality control metrics for the whole-exome sequencing of IgAN patient samples are listed in Table A2. Sex chromosome karyotype and results of analysis of the SRY gene coverage coincided with the data from the patient medical records.

### 2.4. Exome-Wide Association Study of IgAN

**Figure 2:**
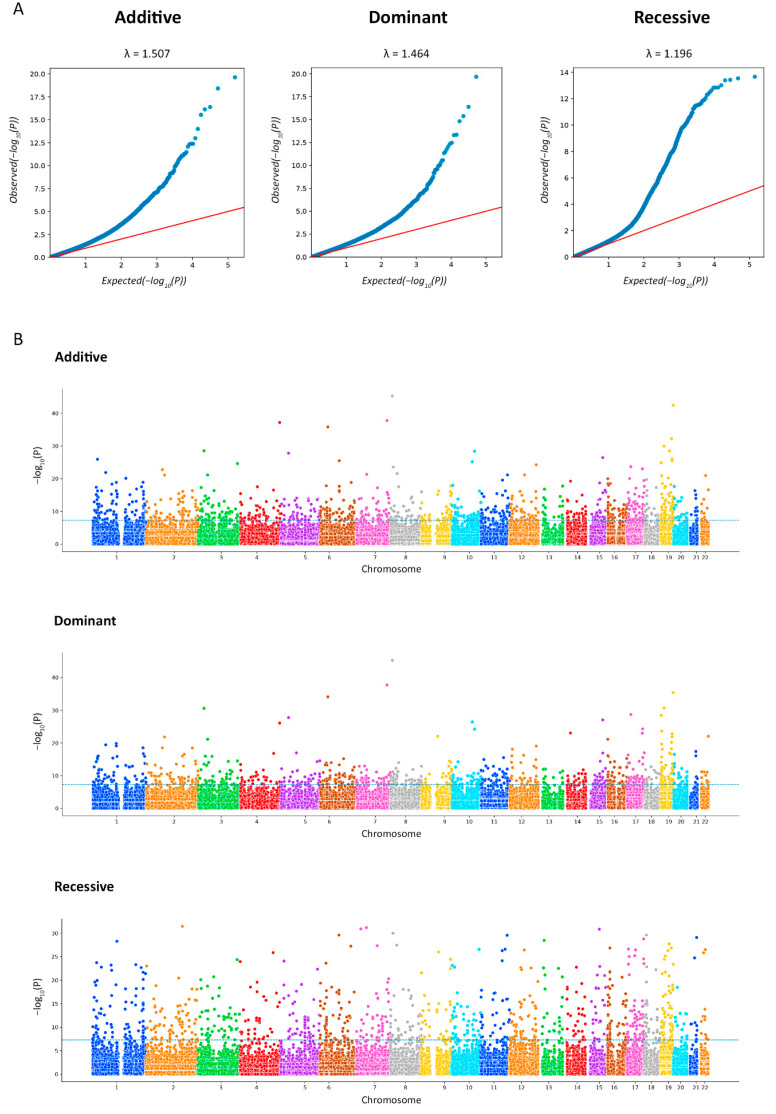
The Q-Q plots for association analysis. Each figure shows the expected (x-axis) and observed (y-axis) log (*p*-values) (**A**). The Manhattan plots for the exome-wide association study (EWAS) of IgA nephropathy (IgAN). The figure shows the *p*-value for the association with the disease (expressed as a negative logarithm of the *p*-value, y-axis) for each tested marker plotted against the chromosomal position of the markers (x-axis). The blue line at 6 represents the threshold for EWAS (−log_10_(1 × 10^−6^)) (**B**).

IgAN patients were successfully matched with control subjects in the case of the recessive model, as clearly shown by the small systematic deviation (λ = 1.196) of the observed distribution from the expected distribution under the null hypothesis which claimed the absence association in the quantile–quantile (Q-Q) plot (Figure 2A). The association *p*-value of multiple markers located on autosomes are shown on the Manhattan plots (Figure 2B).

A total of 333 markers were identified after the *p*-value correction, including 52 insertions/deletions. For each model, we showed the 10 most significant markers, with 24 of them not overlapping (Table 1).

There were 28 multiallelic genetic variants in total (each variant was considered a heterozygote); after the quality procedure, 4 variants remained: chr8:14095250A > T (rs1478030), chr8:14095250A > AT (rs35876033) in gene *SGCZ* (in the first case the minor allele was A; in the second it was AT), as well as chr17:73208205G > T (rs8075486) and chr17:73208205G > GTGTT (rs56406015) in gene *NUP85* (in the first case, the minor allele was T; in the second it was GTGTT, for which the missing genotype count value was 12). Only rs56406015-GTGTT was more common in patients (49.26% vs. 32.78%, *p* = 0.0001; OR 1.992 [95% CI, 1.395–2.844]), but was not statistically significant in logistic association models.

A total of 751 pairwise calculations with r^2^ > 0.2 and 30 with r^2^ > 0.7 were found (Table A3), among which only four markers were significant higher in both the additive and recessive models compared with the controls, and twenty-six were significant only in the recessive model. In total, we detected 86 unique markers with r^2^ > 0.7, that were more frequently found in patients. Moreover, in just one pairwise were there linked markers located in two different genes: rs1695213 (*KRBA2*) and rs422679 (*RPL26*). It is known that the *RPL26* gene is associated with Diamond–Blackfan anemia 11, in which unilateral renal agenesis occurs [39]. The largest number of linked genetic variants (each haplotype not exceeding 200 kb) was in the region chr17:41204856-41361960, where 89 markers were detected.

### 2.5. Gene Set Enrichment and eQTL Analysis

Using the Metascape tool, we built networks showing the specificities underlying the interactions between genes.

**Figure 3:**
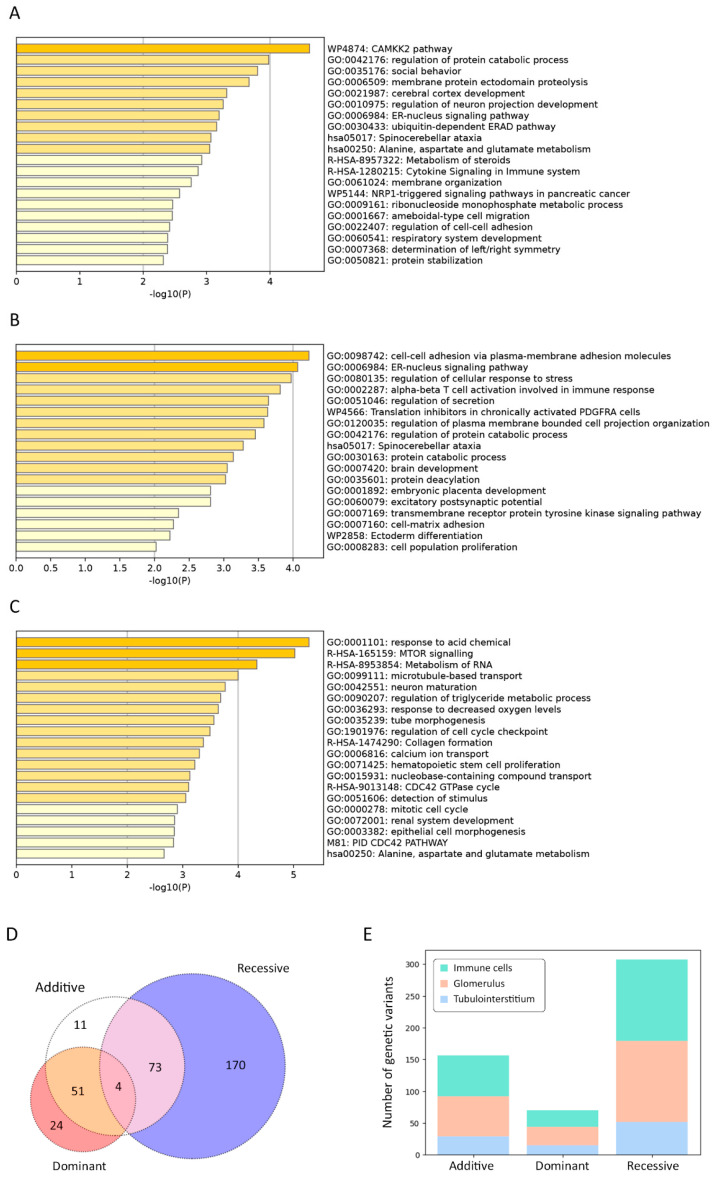
Statistically enriched terms using Metascape, additive (**A**), dominant (**B**), and recessive (**C**) logistic models. The intersection of genetic variant set based on different inheritance models, shown in a Venn diagram (**D**). Markers are interrogated against the following datasets: Database of Immune Cell eQTLs (DICE) project and NephQTL (**E**).

In the additive model, the terms “CAMKK2 pathway” (WP4874) (log[*q*-value] = −0.27) and “positive regulation of catabolic process” (GO:0042176) (log[*q*-value] = −0.15) demonstrated the most significant overexpression (Figure 3A).

In the dominant model, the terms “cell–cell adhesion via plasma-membrane adhesion molecules” (GO:0098742) (log[*q*-value] = −0.19) and “ER–nucleus signaling pathway” (GO:0006984) (log[*q*-value] = −0.19) were significantly more enriched (Figure 3B).

In the recessive model, the most overrepresented terms included “response to acid chemical” (GO:0001101) (log[*q*-value] = −0.97) and “MTOR signaling” (R-HSA-165159) (log[*q*-value] = −0.97) (Figure 3C).

Moreover, cell type signature analysis revealed kidney-related terms to be the topmost. For instance, the “lake adult kidney c5 proximal tubule epithelial cells stress inflame” (M39224) was the first and the second hit for the recessive and additive models, respectively; for the recessive model, the second hit was “lake adult kidney c26 mesangial cells” (M39245). In the case of the dominant model, the least significantly enriched terms were “lake adult kidney c11 thin ascending limb” (M39230) and “lake adult kidney c15 connecting tubule” (M39234). 

For each inheritance model, we detected four variants: rs10710110 (*NBPF3*), rs34527214 (*CYTH2*), rs4803184 (*FBXO27*), and rs71185698 (*PSMD2*) (Figure 3D). Based on the eQTL analysis relying on the NephQTL database, there was no substantial difference between the number of variants affecting the expression in the glomeruli and tubulointerstitium (Figure 3E); however, their number was nearly two times higher than that detected with DICE. We found only two variants in the Kidney eQTLs Atlas: rs2242954 affected *PWP2* expression in renal tubules and *C21orf33* expression in the renal tubules and glomeruli, while rs4803184 influenced *FBXO27* expression in the glomeruli. rs2242954 is also known to elevate the *C21orf33* expression in macrophages (beta = 18.259 ± 4.087, *p* = 1.156 × 10^−5^), NK cells (beta = 9.086 ± 1.943, *p* = 4.593× 10^−6^), CD4+ T cells (beta = 5.722 ± 1.312, *p* = 1.822 × 10^−5^), dendritic cells (beta = 4.964 ± 0.769, *p* = 4.818 × 10^−10^), and CD8+ T cells (beta = 3.611 ± 0.689, *p* = 3.245 × 10^−7^).

### 2.6. Protein–Protein Interaction (PPI) Network Analysis of Potential Targets

**Figure 4:**
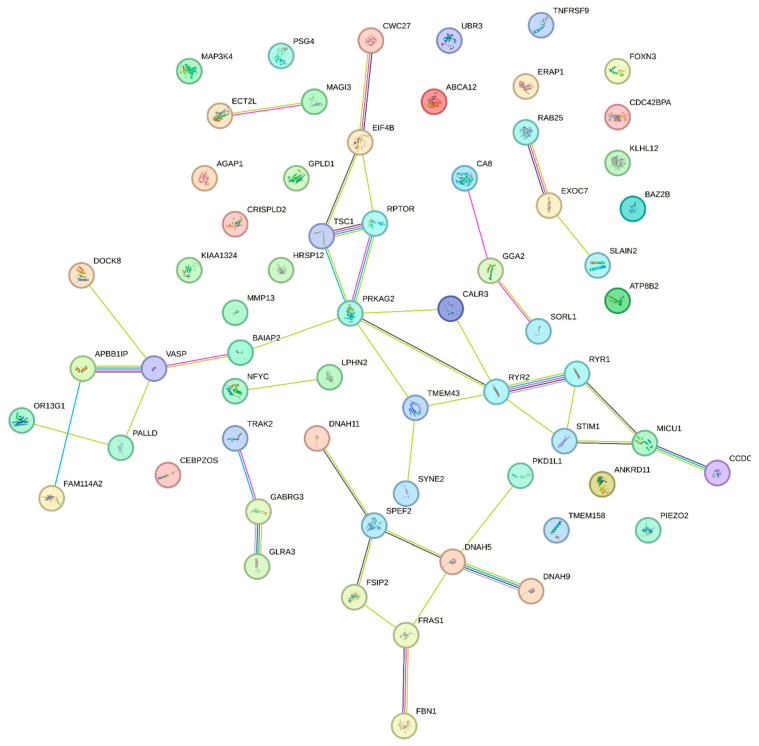
The STRING protein–protein interaction network based on gene interactions, recessive model, one of the three clusters.

The PPI networks were obtained using the String online platform. None of the networks had PPI enrichment *p* < 0.05, the lowest value belonging to the recessive model with *p* = 0.0613. Its network contained 205 nodes, 160 edges, and an average node degree equal to 1.56. After clustering with k-means into three clusters, the most enriched one was the cluster of the recessive model with *p* = 3.09 × 10^−14^ (average node degree was 1.32) (Figure 4).

### 2.7. Transcription Factor (TF) Binding Sites’ Enrichment Analysis

We identified numerous regions with predicted TF binding sites near SNVs that could affect gene expression (Table A4). Eleven SNVs enhance and two SNVs disrupt transcription factor binding sites.

### 2.8. Frequency and Comparison of Allele Frequencies between IgAN Patients and Healthy Donors

Table A5 shows the most frequent HLA alleles in the IgAN patient samples. Among them, we detected 142 unique alleles: 22 *HLA-A*, 34 *HLA-B*, 22 *HLA-C*, 28 *HLA-DRB1*, 7 *HLA-DQA1*, 13 *HLA-DQB1*, and 16 *HLA-DPB1* alleles. The loci *HLA-C* (expected heterozygosity = 92.41%, observed heterozygosity = 90.00%, *p* = 0.0036) and *HLA-DRB1* (expected heterozygosity = 92.65%, observed heterozygosity = 84.29%, *p* = 0.0013) exhibited statistically significant deviations from the Hardy–Weinberg equilibrium (Table A6). In the healthy donors’ group, the most frequent alleles were HLA-A*02:01:01G (27.13%), HLA-B*07:02:01G (11.79%), HLA-C*07:02:01G (13.22%), HLA-DRB1*07:01:01G (13.36%), HLA-DQA1*05:01:01G (24.64%), HLA-DQB1*03:01:01G (22.12%) and HLA-DPB1*04:01:01G (44.19%). Allele HLA-B*56:01:01G (OR, 4.331 [95% CI: 1.739–9.437]) demonstrated the strongest association with IgAN, although it did not reach statistical significance. In the Russian population, this allele was observed only among 1.5% of Nizhniy Novgorod citizens [40]. Alleles HLA-C*01:02:01G (*p* = 0.029; OR, 2.727 [95% CI, 1.477–4.738]), HLA-DRB1*01:01:01G (*p* = 0.034; OR, 2.189 [95% CI, 1.373–3.389]), HLA-DQA1*01:01:01G (*p* = 0.0076; OR, 2.021 [95% CI, 1.322–3.048]), and HLA-DQB1*05:01:01G (*p* = 0.01; OR, 2.124 [95% CI: 1.383–3.195]) were significantly more frequent among IgAN patients.

## 3. Discussion

Our study is the first Russian EWAS conducted on children with IgAN that employs the same population-based control cohort and takes insertions/deletions into consideration as well as utilizes HLA typing with up to 3-field resolution. The limitation of the study is the relatively small patient cohort size, despite applying the strict Bonferroni criterion and high frequency of a minor allele.

Epidemiological data on the frequency of IgAN in Russia are scarce. According to the statistics from the period of 1999–2019, IgAN was the most common type of immune glomerulopathies (41.5%). It was detected in one out of every four kidney biopsies, indicating a higher prevalence compared to Asia, Europe, and America [41]. The annual incidence of IgAN is approximately 8–10 cases per 1 million, and the average age of onset is 34 ± 12 years. Therefore, the size of the children cohort prospectively recruited in Moscow for 3 years aligns with the expected size. 

The most important signals were detected in the loci that had not been described before in IgAN patients. However, through eQTL analysis, we discovered that the variants affect gene expression in the glomeruli and renal tubules and confirmed their connection to significant Metascape categories related to immunity and kidney development. Moreover, the functional enrichment analysis revealed signaling pathways associated with nervous system development, which surprisingly had been already alleged. *ANKRD16* was suggested as a candidate gene for IgAN in the Korean population [42], although its mutant protein was reported to be associated with Purkinje cell degeneration [43] and had not been linked to renal disorders before. One of the examples that might help explain these findings is the *CYLD* gene. Its gain-of-function mutations was observed in Alzheimer’s and Parkinson’s patients, while its loss-of-function mutations were discovered in patients with benign skin neoplasms, with no observed overlap between mutant phenotypes [44]. Another possible explanation is phenotypic heterogeneity. We obtained results comparable to the global data only by studying the HLA loci [17,34]. In the patient group, we identified a slight predominance of the *HLA-DRB1∗03:01:01* [26] and HLA-DQB1*03:01 [28] alleles, although it was not statistically significant. HLA-C*01:02:01 was observed in significantly high frequencies in the Asian populations (up to 19.0%), whereas the frequencies were lower in the Caucasian populations (up to 4.0%) [45]. HLA-C*01:02 is associated with psoriatic arthritis in the population of southern Han Chinese patients [46]. Individuals with HLA-C*01:02:01 may have an increased level of HLA-C expression, which is disadvantageous in inflammatory diseases such as Crohn’s disease [47]. Henoch–Schönlein purpura (HSP) in a population from Northwest Spain is significantly associated with HLA-DRB1*01, with 20% of the patients studied having persistent renal sequelae [48]. Moreover, haplotype DQA1*01:01/DQB1*05:01/DRB1*01:01 did not occur in IBD patients and occurred in 43.5% of patients from Finland with childhood-onset (<17 years) HSP, but not with the severity of the kidney involvement [49]. HSP and IgAN are inflammatory conditions that share pathophysiological mechanisms; however, genes affecting the mucosal immune defense may not be the central defect in the pathogenesis of HSP. In other words, the association of HSP with IBD is unconvincing [50].

rs143409664 is a variant of uncertain significance, since it produces an elongated protein while the alteration in the nucleotide sequence is not located in a repeat region. In the Russian population, the rs143409664-C allele has frequencies of 0.5211 among healthy individuals and 0.5242 among patients, according to RuSeq [51]. *PRAG1* knockout in mice is lethal [52], and this gene has been described in several papers. Although its expression was detected in kidneys as well [53], there is no reliable evidence supporting the association of *PRAG1* with the disease. The frequency of the rs5828672-C variant in the *ZNF787* gene is 0.9085; however, it was absent in health individuals in RuSeq, which may indirectly imply the inexplicable predominance of the allele in IgAN patients. This variant did not show any linkage disequilibrium as well. *ZNF787* has been characterized to a certain extent; it is known to be able to suppress neuron growth and differentiation from induced pluripotent stem cells [54]. The frequency of the *UBR3* gene rs13028230-G allele is 0.639, according to gnomAD, indicating the lower representation of the A allele; however, no information on it could be found in RuSeq. The highest value of r^2^, the indicator of the linkage disequilibrium, in Table 1 belonged to rs72891954 (0.327), which slightly deviates from the value in the global population (0.282, *p* < 0.0001) [55]. Therefore, the hypothesis suggesting that the *UBR3* allele belongs to a haplotype due to population shifts does not appear convincing. The *UBR3* mRNA levels in patients with lupus nephritis correlated with SLEDAI-2K and the index of histological activity [56]. The repertoires of the B and T cell receptors were studied in IgAN patients, and some types were found to be associated with the disease [57]. We identified the previously undescribed variant rs767448033 in the *TRBV5-4* gene. The expression of the transcription factor EOMES was higher in the circulating CD8 T cells (59%) compared to CD4 T cells (15%); however, in the kidney tissue, its expression in CD4 T cells was higher than in blood (32%) [58]. An elevated SREBF1 expression was detected in patients with chronic renal disease [59]. The *CHRNA3* gene variants correlated with eGFR, albumin–creatinine ratio, and albuminuria [60]. Although the *OR10G6* gene is related to the olfactory system, its variant rs1453654 was associated with the elevated expression of IFNγ after smallpox vaccination [61]. In a rat model of hypertension disease, Piezo2 activation was observed in mesangial cells, renin cells, and perivascular mesenchymal cells, implying its contribution to renal fibrosis [62]. Based on the dominant model, we identified the *ITGAM* gene among the IgAN candidate genes with repeatedly confirmed association [63]. Its variant rs60662530 had P_BONF_ = 0.046; however, the frequency ratio did not support the suggestion that the -CTTG allele was more frequent among patients (OR, 2.887 [95% CI: 0.1893–1.992]), as the lower CI was less than 1. Comparing with an exome-wide study in Han Chinese samples in 2021, we found only the rs40986 variant (*FBXL21*), which passed our quality thresholds but did not show significant differences with the control group (*p* = 0.8258, OR, 0.9569 [95% CI: 0.6466–1.416]); the frequency of minor allele -C in patients was 27.14% and in controls was 28.02% [64].

We did not study the external risk factors; therefore, we can rely only on the ratio of the allele frequencies and genotypes from the samples.

Although there are quite a few studies on the genetics of IgAN patients, the role of particular genes as well as signaling pathways in the disease pathogenesis remains elusive. This complexity may be attributed to the highly heterogeneous interactions of hereditary factors or ethnospecificity of their manifestation. Answering these questions require further research on larger samples, utilizing more powerful molecular and genetic techniques such as GWAS and transcriptome comparison.

## 4. Materials and Methods

### 4.1. Ethics Statement

All legal representatives as well as patients over 15 y.o. signed the appropriate informed voluntary consent to participate in the study. The local ethics committee of Pirogov Russian National Research Medical University approved this study on the 17th of December 2018 (Protocol No. 181).

### 4.2. Patient Cohort

Between 2019 and 2021, 70 children at the Russian Children’s Clinical Hospital had been observed at the nephrology department for at least 6 months. During laboratory tests, 4 mL of blood was sampled from the cubital vein into a test tube with EDTA for subsequent EWAS. In all patients, the diagnosis of primary IgA nephropathy was confirmed via kidney biopsy and further histological examination with immunofluorescence, electronic, and light microscopy. Glomerular immunofluorescence for IgA, IgM, IgG, C3, C1q, kappa, lambda, and fibrinogen were scored by the intensity of immunostaining: negative (–), trace (+/–), mild (+), moderate (++), and strong (+++) [65].

### 4.3. gDNA Extraction

DNA isolation was performed using the DNeasy Blood and Tissue Kit (Qiagen, Hilden, Germany) according to the manufacturer’s protocol. The extracted DNA was quantified with the Qubit dsDNA BR Assay system (Life Technologies, Carlsbad, CA, USA), and its quality was assessed via 1% agarose gel electrophoresis.

### 4.4. Library Preparation and Enrichment

DNA Libraries were prepared from 500 ng of genomic DNA using the MGIEasy Universal DNA Library Prep Set (MGI Tech, Shenzhen, China) according to the manufacturer’s protocol. DNA fragmentation was performed via ultrasonication using Covaris S-220 (Covaris, Inc., Woburn, MA, USA) with an average fragment length of 250 bp. Whole-exome enrichment of DNA library pools was performed according to a previously described protocol [66] using the SureSelect Human All Exon v7 probes (Agilent Technologies, Santa Clara, CA, USA). The concentrations of DNA libraries were measured using Qubit Flex (Life Technologies, Carlsbad, CA, USA) with the dsDNA HS Assay Kit (Invitrogen, Waltham, MA, USA) following the manufacturer’s protocol. The quality of the prepared libraries was assessed using Bioanalyzer 2100 with the High Sensitivity DNA kit (Agilent Technologies, Santa Clara, CA, USA) according to the manufacturer’s instructions.

### 4.5. Sequencing

The enriched library pools were further circularized and sequenced via paired end sequencing using DNBSEQ-G400 with the DNBSEQ-G400RS High-throughput Sequencing Set PE100 following the manufacturer’s instructions (MGI Tech, Shenzhen, China), with the average coverage of 100× Fastq files generated using the basecallLite software (ver. 1.0.7.84) from the manufacturer (MGI Tech, Shenzhen, China). 

### 4.6. Control Samples

We used exome sequencing data from 637 healthy Russian donors previously processed in our laboratory as a control dataset. To compare HLA allele frequencies, we used data on healthy donors from the Pirogov University register including 1849 individuals.

### 4.7. Raw Sequencing Data Analysis

The quality control of the obtained paired fastq files was performed using FastQC v0.11.9 [67]. Based on the quality metrics, fastq files were trimmed using BBDuk by BBMap v38.96 [68]. Reads were aligned to the indexed reference genome GRCh37 using bwa-mem2 v2.2.1 [69]. SAM files were converted into bam files and sorted using SAMtools v1.9 to check the percentage of the aligned reads [70]. The duplicates in the obtained bam files were marked using Picard MarkDuplicates v2.22.4 [71] and excluded from further analysis.

We performed quality control analysis on marked bam files with the following Agilent all-exon v7 target file “regions.bed”. For the samples that passed quality control (width of target coverage 10× ≥ 95%), single-nucleotide variants (SNVs) and indels were called using the bcftools mpileup software v1.9 [72], and vcf files were obtained for each sample. After variant calling, vcf files were normalized using vt normalize v0.5772-60f436c3 [73] and filtered based on the target regions expanded by ± 100 base pairs flanking each end. Calling data were annotated using the InterVar software (ver. py 2.0.2 20190327) [74].

### 4.8. Exome-Wide Association Study

We analyzed DNA samples from 70 IgAN patients and 637 samples from healthy donors. Variants in vcf files (ver. 4.2) were filtered based on coverage (threshold = 13) and quality (threshold = 20). InDel variants were normalized using vt v0.5772-60f436c3 tool. All vcf files were merged with bcftools merge (ver. 1.10.2). To obtain the list of genes containing sets of false positive variations, the genomic intervals were extracted from the gtf file of genome annotation (GRCh37.p13). Then, using bedtools (ver. 2.27.1) [75], variations in these genomic intervals were excluded from the merged vcf files. Data were analyzed in the PLINK v1.90b6.24 software [76]. The deviation of sample heterozygosity levels was within 3 SD from the mean. The markers (both SNVs and indels) genotyped in less than 98% of the samples, as well as individuals with less than 98% of genotyped markers, were filtered out. In all samples, predicted sex coincided with the actual sex. Due to a small sample size, we chose a threshold of 0.0001 for checking the Hardy–Weinberg equilibrium and a frequency of a minor allele of 0.1. After setting the threshold value PI_HAT = 0.2, we did not exclude samples from the analysis. Finally, 78,020 of 793,015 markers were subjected to further analysis. We created the logistic regression additive, dominant, and recessive genetic models (the covariate was the first principal component); *p*-value was corrected using the Bonferroni criterion (P_adj_ < 0.05). 

### 4.9. Functional Enrichment Analysis and Transcription Factor (TF) Binding Preference Detection

The charts of gene enrichment analysis were created using the Metascape v3.5.20230101 [77]. The eQTL analysis was performed using the following databases: Kidney eQTLs Atlas [78], NephQTL [79], and Database of Immune Cell eQTLs (DICE) project [80]. The cut-off for *p*-value was *p* < 0.05. The STRING (https://string-db.org, date of access 12 April 2023) database was used to predict functional interactions of proteins. The search was restricted to “Homo sapiens” and an interaction score limit of > 0.4 was applied to construct the Protein–Protein Interaction (PPI) networks. We applied atSNP [81] to predict differential binding of a TF to a marker by labeling SNP-motif combinations with atSNP pval_diff < 0.05 as significant.

### 4.10. HLA-Typing

HLA typing was performed on the exome data with help of three programs: HLA-HD (ver. 1.7.0) [82], HLAScan (ver. 2.1.4) [83], and Kourami (ver. 0.9.6) [84] using the allele database IPD-IMGT/HLA (ver. 3.51.0) [85]. The obtained data on the alleles were transformed into the corresponding G-groups, and results were merged into the final version. In case different tools provided conflicting results, we chose the alleles identified using HLA-HD or Kourami and checked bam files in IGV [86].

### 4.11. Statistical Analysis

**Figure A1:**
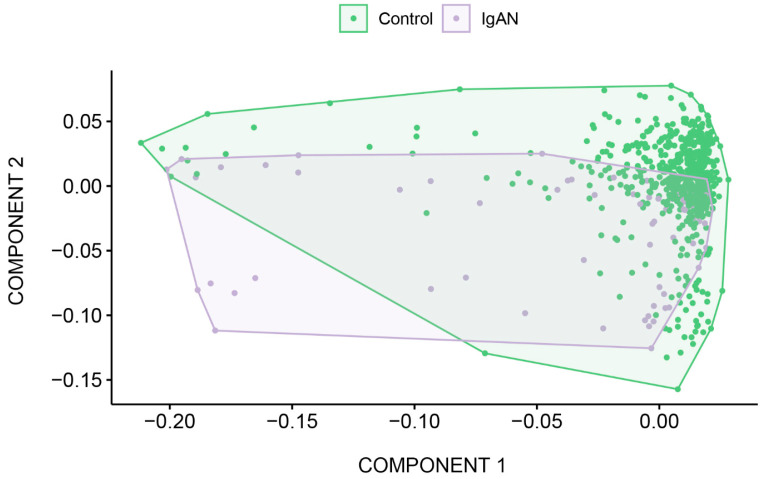
Principal component analysis of the IgA nephropathy samples comprising the samples from healthy donor cohort.

**Figure A2:**
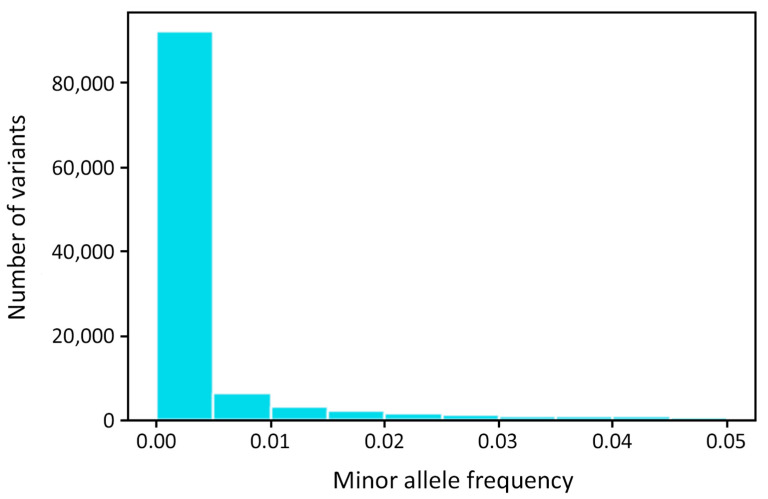
Minor allele frequency distribution in IgA nephropathy patients and healthy donors.

Principal component analysis and allele frequency estimation were performed using PLINK v1.90b6.24 (Figure A1 and Figure A2). Manhattan and Q-Q plots were generated with Python scripts. The one-way or two-way ANOVA test, Chi-squared test, and Fisher’s exact test [87] were used to compare the differences between groups in RStudio 2022.02.0. The multiple comparison correction was conducted using the Bonferroni method: we multiplied *p*-values by the number of the alleles under consideration. Testing for the Hardy–Weinberg equilibrium was performed using the Arlequin 3.5 software [88]. All data were presented as mean ± standard deviation (SD) or median [Q1, Q3]. Data normality was checked using the Kolmogorov–Smirnov test.

## Figures and Tables

**Table 1:** The exome-wide association study analysis of IgAN patients and control groups.

GENE	CHR	POSITION	MARKER	BONF	A1	F_A	F_U	A2	CHISQ	*p*	OR (95% CI)	SE
Additive												
*PRAG1*	8	8,176,387	rs143409664	1.808 × 10^–15^	CGGGGCG	0.4571	0.07692	C	179.8	5.464 × 10^–41^	10.11 (6.834–14.94)	0.1996
*ZNF787*	19	56,599,437	rs5828672	2.926 × 10^–14^	C	0.4929	0.07849	CTCG	205.8	1.13 × 10^–46^	11.41 (7.731–16.84)	0.1986
*TRBV5-4*	7	142,168,890	rs767448033	3.146 × 10^–12^	T	0.3571	0.07849	C	103.3	2.906 × 10^–24^	6.522 (4.365–9.745)	0.2048
*TENM3*	4	183,370,244	rs35591339	5.646 × 10^–12^	A	0.5071	0.08399	AGCG	205.3	1.485 × 10^–46^	11.22 (7.629–16.51)	0.1969
*PIM1*	6	37,138,023	rs1300416314	2.207 × 10^–11^	G	0.4286	0.1148	GGCA	99.66	1.81 × 10^–23^	5.784 (3.969–8.429)	0.1921
*CYTH2*	19	48,985,178	rs34527214	7.812 × 10^–10^	TA	0.5357	0.1546	T	117.9	1.814 × 10^–27^	6.308 (4.378–9.089)	0.1863
*MISP3*	19	14,184,713	rs35666756	8.00 × 10^–9^	CG	0.3429	0.09105	C	78.01	1.028 × 10^–18^	5.208 (3.499–7.753)	0.2029
*EOMES*	3	27,763,427	rs368178421	3.243 × 10^–8^	GCGGCGC	0.3623	0.1009	G	76.89	1.805 × 10^–18^	5.06 (3.418–7.491)	0.2001
*FBXO27*	19	39,505,111	rs4803184	3.255 × 10^–8^	G	0.4786	0.1389	A	101.9	5.818 × 10^–24^	5.688 (3.938–8.216)	0.1876
*SH3PXD2A*	10	105,428,453	rs10533306	3.656 × 10^–8^	C	0.4714	0.1852	CAG	61.51	4.41 × 10^–15^	3.923 (2.735–5.627)	0.184
Dominant												
*PRAG1*	8	8,176,387	rs143409664	1.654 × 10^–15^	CGGGGCG	0.4571	0.07692	C	179.8	5.464 × 10^–41^	10.11 (6.834–14.94)	0.1996
*TRBV5-4*	7	142,168,890	rs767448033	3.146 × 10^–12^	T	0.3571	0.07849	C	103.3	2.906 × 10^–24^	6.522 (4.365–9.745)	0.2048
*ZNF787*	19	56,599,437	rs5828672	3.25 × 10^–11^	C	0.4929	0.07849	CTCG	205.8	1.13 × 10^–46^	11.41 (7.731–16.84)	0.1986
*PIM1*	6	37,138,023	rs1300416314	1.187 × 10^–10^	G	0.4286	0.1148	GGCA	99.66	1.81 × 10^–23^	5.784 (3.969–8.429)	0.1921
*MISP3*	19	14,184,713	rs35666756	3.523 × 10^–9^	CG	0.3429	0.09105	C	78.01	1.028 × 10^–18^	5.208 (3.499–7.753)	0.2029
*EOMES*	3	27,763,427	rs368178421	3.964 × 10^–9^	GCGGCGC	0.3623	0.1009	G	76.89	1.805 × 10^–18^	5.06 (3.418–7.491)	0.2001
*SREBF1*	17	17,740,164	rs60282872	2.629 × 10^–8^	G	0.2643	0.08477	GC	44.17	3.013 × 10^–11^	3.878 (2.537–5.928)	0.2165
*PRTN3*	19	843,692	rs2301879	3.49 × 10^–8^	G	0.2929	0.08948	A	53.45	2.646 × 10^–13^	4.214 (2.792–6.361)	0.2101
*AMACR*	5	34,008,206	rs3217251	6.688 × 10^–8^	C	0.2857	0.08085	CCGGCGCCACGCCCCCAGCCG	58.24	2.322 × 10^–14^	4.548 (2.993–6.91)	0.2135
*CHRNA3*	15	78,913,067	rs751352647	1.363 × 10^–7^	A	0.4214	0.1546	ACAG	60.56	7.149 × 10^–15^	3.982 (2.755–5.755)	0.1879
Recessive												
*UBR3*	2	170,871,976	rs13028230	1.545 × 10^–9^	A	0.5429	0.2951	G	35.59	2.431 × 10^–9^	2.836 (1.991–4.039)	0.1804
*PKD1L1*	7	47,835,116	rs2348459	2.031 × 10^–9^	T	0.5571	0.2363	C	66.2	4.077 × 10^–16^	4.067 (2.844–5.815)	0.1825
*DNAH11*	7	21,639,818	rs6461586	2.649 × 10^–9^	T	0.3643	0.1586	C	36.34	1.657 × 10^–9^	3.041 (2.089–4.427)	0.1916
*CIAO2A*	15	64,381,148	rs16947748	2.861 × 10^–9^	G	0.5786	0.2841	A	50.83	1.01 × 10^–12^	3.459 (2.421–4.942)	0.1821
*TRMT9B*	8	12,870,438	rs2466264	6.754 × 10^–9^	C	0.6714	0.3265	G	65.07	7.224 × 10^–16^	4.215 (2.907–6.112)	0.1896
*MDN1*	6	90,400,292	rs954638	9.888 × 10^–9^	C	0.4929	0.179	T	74.9	4.943 × 10^–18^	4.458 (3.108–6.397)	0.1842
*PIEZO2*	18	10,757,868	rs7242408	1.003 × 10^–8^	A	0.5071	0.2276	G	51.83	6.039 × 10^–13^	3.491 (2.445–4.986)	0.1818
*OR10G6*	11	123,865,086	rs7944434	1.053 × 10^–8^	A	0.4571	0.1743	C	62.56	2.583 × 10^–15^	3.991 (2.777–5.735)	0.185
*PWP2*	21	45,547,563	rs2242954	1.658 × 10^–8^	G	0.4	0.1931	C	32.15	1.427 × 10^–8^	2.786 (1.933–4.016)	0.1865
*BAIAP2*	17	79,084,072	rs4072588	2.268 × 10^–8^	T	0.5214	0.2339	G	53.88	2.132 × 10^–13^	3.568 (2.499–5.095)	0.1817

Notes: CHR—chromosome, POSITION—base pair based on the human reference genome GRCh37, A1—minor allele, F_A—frequency of A1 allele in patients, F_U—frequency of A1 allele in controls, A2—major allele, CHISQ—basic allelic test chi-square (1df), *p*—*p*-value for CHISQ, OR (95% CI)—odds ratio and confidence interval for odds ratio, SE—standard error, BONF—Bonferroni single-step adjusted *p*-values.

## Data Availability

The data underlying this article will be shared on reasonable request to the corresponding author.

## Appendix A

**Table A1:** Clinical characteristics of the IgA nephropathy patients.

Features		(n = 70)
Sex	Male	64.4%
	Female	35.6%
Age of the onset, years		10.3 ± 3.8
Serum creatinine level (mg/dL)		46.5 ± 7.1
eGFR (mL/min/1.73m^2^)		97.7 ± 26.9
Proteinuria (g/day/1.73m^2^)		0.9 [0.5–2.0]
Mean blood pressure (mm Hg)		84.5 ± 12.42
Symptoms at the debut	persistent microscopic hematuria	20.0%
	macroscopic hematuria	38.6%
	nephritic syndrome	11.5%
	nephrotic syndrome	25.7%
	acute kidney injury	2.8%
	rapidly progressive glomerulonephritis	1.4%

Notes: Conversion factors for units: serum creatinine in mg/dL to μmol/L, ×88.4.

**Table A2:** Key quality control metrics for exome sequencing of the experimental samples from the IgA nephropathy cohort.

Metrics	Mean	Min	Max
Single reads per sample	79,494,574	41,389,840	162,054,108
Estimated library size	167,316,756	43,904,883	362,437,565
Duplicates	12.33	5.86	26.82
On-target bases	85.3%	62%	92%
Mean target coverage	89.65	46.2	140
Median target coverage	80.62	42	128
Width 10×	96.9%	94.3%	97%
Width 20×	95.2%	85%	97%
Width 30×	91.2%	70%	96%

**Table A3:** The genetic markers with r^2^ > 0.7, that were significant in at least one logistic regression model.

Gene	MARKER	CHR	BP	A1	F_A	F_U	A2	*p*	OR (95% CI)	BONF ADD	BONF DOM	BONF REC	R^2^
*LGALS8*	rs2244809	1	236,702,117	T	0.55	0.2849	G	1.274 × 10^–10^	3.067 (2.152–4.372)	0.01239	ns	2.732 × 10^–5^	0.9507 (rs1041937); 0.865722 (rs2244808); 0.660795 (rs11807205)
	rs2244808		236,702,153	G	0.6	0.3163	A	2.016 × 10^–11^	3.242 (2.266–4.638)	0.007593	ns	1.236 × 10^−4^	0.865722 (rs2244809); 0.822342 (rs1041937); 0.759458 (rs11807205)
	rs1041937		236,702,210	A	0.4857	0.2857	G	1.08 × 10^–6^	2.361 (1.659–3.361)	ns	ns	0.01863	0.822342 (rs2244808); 0.9507 (rs2244809); 0.668001 (rs11807205)
	rs11807205		236,702,475	A	0.6286	0.3501	G	1.106 × 10^–10^	3.142 (2.188–4.511)	0.02077	ns	8.544 × 10^−4^	0.759458 (rs2244808); 0.660795 (rs2244809); 0.668001 (rs1041937)
	rs2254823		236,711,297	A	0.55	0.2849	G	1.274 × 10^–10^	3.067 (2.266–4.638)	0.007593	ns	1.236 × 10^−4^	0.96159
	rs15701		236,711,323	T	0.6	0.3163	A	2.016 × 10^–11^	3.242 (1.855–3.761)	ns	ns	0.02441	
*COX7A2L*	rs12613284	2	42,562,787	C	0.5071	0.3462	G	1.685 × 10^−4^	1.944 (1.368 –2.761)	ns	ns	0.043	0.991318
	rs10194796		42,562,821	A	0.5071	0.3454	C	1.555 × 10^−4^	1.95 (1.373–2.77)	ns	ns	0.03463	
*DNAH5*	rs6554808	5	13,735,147	C	0.6	0.3666	A	7.614 × 10^−8^	2.592 (1.814–3.703)	ns	ns	3.987 × 10^−4^	0.85481
	rs2401811		13,737,700	A	0.5714	0.3611	G	1.157 × 10^–6^	2.359 (1.656–3.361)	ns	ns	0.006805	
*MYL10-CUX1*	rs740202	7	101,280,652	T	0.5929	0.4074	G	2.52 × 10^–5^	2.118 (1.485–3.022)	ns	ns	0.006267	0.997391
	rs1636453		101,281,232	A	0.5929	0.4082	T	2.737 × 10^–5^	2.111 (1.48–3.012)	ns	ns	0.007734	
*OR4K3*	rs5807006	14	20,336,681	A	0.5429	0.2504	AG	2.464 × 10^–13^	3.555 (2.491–5.074)	ns	ns	0.007604	0.734943
	rs1892239	14	20,336,733	A	0.45	0.2206	G	1.905 × 10^–9^	2.891 (2.021–4.137)	ns	ns	0.00812	
*OR4E1*	rs7144135	14	22,138,328	G	0.45	0.1915	A	1.905 × 10^–12^	3.454 (2.407–4.956)	ns	ns	9.527 × 10^−4^	0.996356
	rs970025	14	221,384,37	A	0.45	0.1907	G	1.538 × 10^–12^	3.471 (2.419–4.981)	ns	ns	6.463 × 10^−4^	
*SYNE2*	rs10138253	14	64,520,416	G	0.3786	0.1586	A	1.301 × 10^–10^	3.233 (2.226–4.695)	ns	ns	9.15 × 10^–6^	0.870958
	rs1255972	14	64,532,107	A	0.3429	0.1633	G	1.621 × 10^–7^	2.674 (1.83–3.907)	ns	ns	0.001809	
*FBN1*	rs11070641	15	48,701,029	C	0.4	0.2221	T	2.882 × 10^–6^	2.335 (1.624–3.356)	ns	ns	6.838 × 10^−4^	0.871057
	rs4775760	15	48,701,612	A	0.4357	0.2386	C	4.227 × 10^–7^	2.464 (1.722–3.525)	ns	ns	8.167 × 10^−4^	
*LOC400499*	rs9932266	16	11,594,747	G	0.5214	0.3053	T	2.285 × 10^–7^	2.479 (1.743–3.526)	ns	ns	2.209 × 10^–5^	0.860027
	rs4781105	16	11,596,040	C	0.5071	0.3148	G	4.606 × 10^–6^	2.24 (1.576–3.185)	ns	ns	1.181 × 10^−4^	
*CRISPLD2-ZDHHC7*	rs2646133	16	84,953,145	A	0.4929	0.3383	G	2.826 × 10^−4^	1.901 (1.338–2.700)	ns	ns	0.03866	0.739664 (rs2646130); 0.988681 (rs2646132); 0.988681 (rs60944306)
	rs2646132	16	84,953,232	C	0.4929	0.3367	T	2.417 × 10^−4^	1.914 (1.347–2.719)	ns	ns	0.031	1 (rs60944306); 0.988681 (rs2646133); 0.747952 (rs2646130)
	rs60944306	16	84,953,233	A	0.4929	0.3367	C	2.417 × 10^−4^	1.914 (1.347–2.719)	ns	ns	0.031	0.747952 (rs2646130); 1 (rs2646132); 0.988681 (rs2646133)
	rs2646130	16	84,953,351	G	0.4571	0.2645	A	1.59 × 10^–6^	2.341 (1.642–3.339)	ns	ns	0.002581	0.747952 (rs2646132); 0.739664 (rs2646133); 0.747952 (rs60944306)
*KRBA2*	rs1695213	17	8,279,324	T	0.6	0.3344	C	5.215× 10^–10^	2.986 (2.088–4.27)	ns	ns	1.967 × 10^–7^	0.907883
*RPL26*	rs422679	17	8,285,398	T	0.6214	0.3579	C	1.166 × 10^–9^	2.945 (2.054–4.222)	ns	ns	4.826 × 10^–6^	
*CEP192*	rs9783903	18	13,000,210	A	0.5429	0.3595	G	2.159 × 10^–5^	2.116 (1.488–3.007)	ns	ns	0.01879	0.976515
*CEP192*	rs1787008	18	13,058,318	A	0.5357	0.354	G	2.418 × 10^–5^	2.106 (1.482–2.992)	ns	ns	0.02432	

Notes: CHR—chromosome, BP—base pair based on the human reference genome GRCh37, A1—minor allele, F_A—frequency of A1 allele in patients, F_U—frequency of A1 allele in controls, A2—major allele, *p*—*p*-value for CHISQ, OR (95% CI)—odds ratio and confidence interval for odds ratio, BONF—Bonferroni single-step adjusted p-values (ADD—additive, DOM—dominant and REC—recessive logistic models), R^2^—intervariant allele count squared correlations. ns—not significant.

**Table A4:** The TF binding motifs affected by potentially deleterious variants.

SNP	TF	Motif	TF Binding Site	*p*
rs3087873	SMAD	AP1_disc10	Enhance	0.00212
rs3087873	ESRRA	AP1_disc10	Enhance	0.00212
rs3087873	MYC	AP1_disc10	Enhance	0.00212
rs2286239	RAD21	RAD21_disc5	Enhance	0.0034
rs33749	ETS	RAD21_disc5	Enhance	0.00348
rs622861	REST	RUNX2_1	Enhance	0.00414
rs2209956	AP1	HLTF_1	Enhance	0.00858
rs11807205	SOX10	LEF1_1	Enhance	0.01723
rs11807205	TFAP2A	LEF1_1	Enhance	0.01723
rs11807205	GATA3	LEF1_1	Enhance	0.01723
rs11807205	E2F1	LEF1_1	Enhance	0.01723
rs11807205	SETDB1	LEF1_1	Enhance	0.01723
rs12495243	EN1	EN1_2	Enhance	0.01832
rs2042367	LEF1	GATA_1	Enhance	0.02353
rs33749	ZNF143	CHD2_disc3	Enhance	0.02499
rs4535042	TFAP2A	TFAP2A_1	Enhance	0.03305
rs4535042	TFAP2A	TFAP2A_4	Enhance	0.03305
rs12495243	VDR	VDR_1	Disrupt	0.03416
rs3087873	PITX2	REST_disc8	Enhance	0.03947
rs3087873	ELF1	REST_disc8	Enhance	0.03947
rs3087873	GATA2	REST_disc8	Enhance	0.03947
rs913178	VDR	EP300_disc9	Disrupt	0.0429
rs913178	AP1	EP300_disc9	Disrupt	0.0429
rs913178	BCL	EP300_disc9	Disrupt	0.0429

Notes: TF—transcription factor, Motif—binding sites with high affinity to TF. *p*—*p*-value that statistically confirms potential stimulation or loss of function of the genomic region harboring SNP in terms of TF binding.

**Table A5:** The distribution of the alleles HLA-A, -B, -DRB1, -DQA1, -DQB1 and -DPB1 in IgA nephropathy (IgAN) patients and healthy donors.

Allele	IgAN (%)	Control (%)	*p*’ (chi-sq)	*p*’ (Fisher’s Exact Test)	OR	95% CI
*A*02:01:01G*	30.00	27.13	>0.05		1.151	0.777–1.682
*A*24:02:01G*	16.43	10.98	>0.05		1.594	0.961–2.543
*A*01:01:01G*	9.29	12.14	>0.05		0.741	0.381–1.327
*A*03:01:01G*	8.57	13.95	>0.05		0.578	0.289–1.055
*A*31:01:02G*	5.71	2.00		>0.05	2.967	1.210–6.327
*B*35:01:01G*	9.29	6.35	>0.05		1.508	0.770–2.725
*B*27:05:02G*	7.14	3.87	>0.05		1.912	0.877–3.734
*B*56:01:01G*	5.71	1.38		>0.05	4.331	1.739–9.437
*B*51:01:01G*	5.71	5.19	>0.05		1.107	0.461–2.291
*B*07:02:01G*	5.71	11.79	>0.05		0.454	0.190–0.929
*C*04:01:01G*	16.43	12.41	>0.05		1.387	0.837–2.210
*C*01:02:01G*	11.43	4.52	0.029		2.727	1.477–4.738
*C*02:02:02G*	10.00	6.06	>0.05		1.723	0.901–3.0621
*C*06:02:01G*	9.29	11.95	>0.05		0.754	0.387–1.351
*C*03:04:01G*	6.43	5.16	>0.05		1.261	0.556–2.521
*DRB1*01:01:01G*	40.00	10.25	0.034		2.189	1.373–3.389
*DRB1*03:01:01G*	9.29	7.73	>0.05		1.221	0.625–2.120
*DRB1*13:01:01G*	8.57	6.92	>0.05		1.260	0.626–2.319
*DRB1*07:01:01G*	7.86	13.36	>0.05		0.553	0.267–1.033
*DRB1*11:04:01G*	6.43	4.87	>0.05		1.343	0.591–2.686
*DQA1*05:01:01G*	30.00	24.64	>0.05		1.310	0.869–1.949
*DQA1*01:01:01G*	28.57	16.51	0.0076		2.021	1.322–3.048
*DQA1*03:01:01G*	17.86	11.40	>0.05		1.689	1.012–2.733
*DQA1*01:03:01G*	10.00	9.13	>0.05		1.105	0.567–2.009
*DQA1*02:01:01G*	7.86	13.41	>0.05		0.551	0.262–1.048
*DQB1*03:01:01G*	26.43	22.12	>0.05		1.265	0.837–1.875
*DQB1*05:01:01G*	24.29	13.12	0.01		2.124	1.383–3.195
*DQB1*02:01:01G*	14.29	18.39	>0.05		0.740	0.433–1.204
*DQB1*06:03:01G*	8.57	7.17	>0.05		1.214	0.603–2.233
*DQB1*03:02:01G*	7.14	7.22	>0.05		0.988	0.458–1.906
*DPB1*04:01:01G*	32.14	44.19	>0.05		0.598	0.408–0.868
*DPB1*04:02:01G*	22.14	13.44	>0.05		1.831	1.174–2.786
*DPB1*02:01:02G*	15.71	14.66	>0.05		1.086	0.649–1.740
*DPB1*03:01:01G*	10.00	9.84	>0.05		1.018	0.535–1.795
*DPB1*06:01:01G*	3.57	1.43		>0.05	2.546	0.782–6.470

Notes: chi-sq—basic allelic test chi-square (1df), OR—odds ratio for Fisher’s exact test and basic allelic test chi-square, 95% CI—odds ratio and confidence interval for odds ratio for Fisher’s exact test and basic allelic test chi-square, *p*’—Bonferroni single-step adjusted *p*-values.

**Table A6:** Conformity to Hardy–Weinberg equilibrium (IgA nephropathy patients).

Locus	No. of Genotypes	Observed Heterozygosity	Expected Heterozygosity	*p*-Value
*HLA-A*	70	0.88571	0.86228	0.41045
*HLA-B*	70	0.92857	0.96310	0.18927
*HLA-C*	70	0.90000	0.92405	0.00358
*HLA-DRB1*	70	0.84286	0.92652	0.00125
*HLA-DQA1*	70	0.67143	0.78335	0.05311
*HLA-DQB1*	70	0.72857	0.83659	0.09385
*HLA-DPB1*	70	0.78571	0.81408	0.18547
